# Exploring the core competencies of clinical nurses in chinese tertiary hospitals: a qualitative content analysis

**DOI:** 10.1186/s12912-023-01337-2

**Published:** 2023-05-17

**Authors:** Meihan Chen, Aiping Wang, Baosen Zhou

**Affiliations:** 1 Department of Public Utility, the First Affiliated Hospital of China Medical University, No.155, Nanjing North Street, Heping District, Shenyang, Liaoning Province China; 2 Department of Clinical Epidemiology and Evidence-Based Medicine, the First Affiliated Hospital of China Medical University, No.155, Nanjing North Street, Heping District, Shenyang, Liaoning Province China

**Keywords:** Nurse, Tertiary hospital, The onion model, Core competency

## Abstract

**Background:**

With the changes in social and medical environments and people’s health needs, the nursing core competency should be updated and developed promptly. This study aimed to explore the core competencies of nurses in Chinese tertiary hospitals under the new health development strategy.

**Methods:**

Descriptive qualitative research was conducted using qualitative content analysis. 20 clinical nurses and nursing managers from 11 different provinces and cities were interviewed via purposive sampling.

**Results:**

Data analysis revealed 27 competencies, which were grouped into three major categories according to the onion model. These categories were motivation and traits (responsibility, enterprise, etc.), professional philosophy and values (professionalism, career perception, etc.), and knowledge and skills (clinical nursing competency, leadership and management competency, etc.).

**Conclusion:**

Based on the onion model, core competencies for nurses in Chinese tertiary hospitals were established, revealing three layers of core competencies and giving a theoretical reference for nursing managers to conduct competency training courses based on the competency levels.

## Background

Core competency is defined as the essential minimum set of attributes, such as applied knowledge, skills, and attitudes, that enable an individual to perform a set of tasks effectively and follow appropriate standards [1]. Core competencies serve as a common language for all healthcare professions, defining what all people are expected to be able to do to the best of their abilities. There is currently no international standard for defining the concept of nursing core competency (NCC), the International Nurses Association and individual national nursing associations define core competencies for nurses differently based on nursing practice and nursing developments in their respective countries [2–7]. The NCC has been defined by international nursing associations and national nursing associations by national nursing practice and developments. As nurses struggle to adapt to standard definitions of competency, nursing practice and public expectations continue to change, and these factors make defining competencies in practice an ongoing challenge [8]. NCC, while defined differently, must be an integrated and dynamically evolving competency. It combines multiple competencies such as knowledge, skills, talents, and personal characteristics and should be updated as a society, the healthcare environment, and the nursing profession change. Because of an aging population and changes in the disease spectrum, the healthcare environment has changed dramatically. Simultaneously, as medical care improves, people’s health needs and concepts evolve, and people are increasingly concerned not only with their physical health, but also with spiritual, psychological, social, environmental, and other factors [9]. The demand for social health has contributed to the development of nursing competency. The health needs of society have contributed to the development of nursing competency while also raising the bar for the renewal and expansion of nursing competency and roles. Nurses are gradually moving from the subordinate role of physicians to independence [10], they are unstoppable forces shaping global healthcare, providing better care and attention to individuals and families all over the world. In China, nurses’ attempts to enforce prescriptive authority are also enhancing nurses’ abilities to “be at the table”, which is a significant step forward in the renewal of nurses’ competency and roles [11]. Nursing services are being extended from institutions to communities and families through the development of new nursing service models such as “Internet + nursing services,“ with nurses playing an important role in providing professional care, health management, rehabilitation promotion, and other comprehensive nursing services. Nurse competency development must reflect the current state of health care [12] and in an ever-changing world, new competencies are required [13].

Some studies have shown that China has a chronic shortage of nursing human resources and that nurses’ competencies vary, meeting the current social demand for high levels of care more difficult. Chinese nursing managers have long struggled with the issue of how to use effective methods to promote nurse competency development [14].To better seek ways and strategies to enhance nurses’ competency, it is critical to understand the hierarchy of the nurse’s competency needs. Competency models have been used in job analysis, performance management, and human resource training and development as important tools in human resource development [15]. The onion model, a classic competency model, has been widely used in competency assessment and measurement. the onion model categorizes competence into three layers: the inner layer, the middle layer, and the outer layer. The inner layer consists of motivation and characteristics, the middle layer of attitudes, values, and self-image, and the outer layer of knowledge and skills [16]. The inner layer’s competency is less likely to be trained, developed, and measured, whereas the outer layer’s competency is more likely to be acquired and assessed through training.

Although many core competency structures existed in China in the past, with the passage of time and the renewal of society’s health needs, nurses have been assigned new roles and responsibilities in the new health development strategy, and the renewal and development of core competencies for nurses is an unavoidable trend [17]. Because of its rich hierarchical features, the onion model better explains the embodiment of nurses’ core competencies at different levels. This study is based on the general layout of social health strategies and the actual needs of nurses, as well as a qualitative study based on the theoretical foundation of the onion model, which can provide rich theoretical guidance for future CBE for nurses.

## Method

### Design

From June to August 2022, semi-structured interviews with 20 clinical nurses and nursing managers (including nurse leaders and nursing department directors) in 11 Chinese provinces and cities were conducted using descriptive qualitative research. Descriptive research is rooted in existing knowledge, thoughtful linkages with other scholars in the field, and the clinical experience of study members [18, 19]. The collected data were analyzed using content analysis, a design that provides contextual descriptions and explanations of social phenomena while also facilitating an understanding of participants’ voices, perspectives, and ideas [20]. The purpose of this qualitative study was to explore the core competencies of nurses in Chinese tertiary hospitals under the new health development strategy. The report of this study is based on the Comprehensive Criteria for Qualitative Research Report (COREQ) checklist [21].

### Sample and recruitment

To ensure the operationalization of the interview questions, we recruited the sample using purposive sampling based on the requirements of the qualitative studies [22]. Recruitment took place in 11 cities and provinces, including Beijing, Shanghai, Guangdong, Fujian, Hunan, Sichuan, Henan, Shandong, Shaanxi, Jilin, and Liaoning based on the principle of maximum differentiation. These cities and provinces cover the northern, southwestern, central, and eastern parts of China, which can better reflect the capacity characteristics of nurses in different geographic areas. In total, 20 people agreed to take part in our research. Nurses and nurse managers were among those recruited until saturation was reached (no additional information was obtained).

### Study instrument

#### Demographic characteristics of the participants

A questionnaire on demographic factors, including gender, age, title, position, education, years of experience, section, and province was prepared following thorough literature research and rigorous group discussions.

#### Interview outline

Following a literature research and group discussion, an initial interview outline was constructed based on existing knowledge about the subject and the literature review [6, 23–25]. The initial interview outline was developed which focuses on the knowledge, skills, attitudes, and roles that nurses should have under the new health development strategy as the main finding of this study. Subsequently, three participants (both clinical nurse and nurse manager) were recruited for pre-interviews via purposive sampling. Finally, three experts with advanced titles and doctorates in clinical nursing and nursing administration were invited to revise the final interview outline, which is shown in Table 1.

**Table 1:** Interview outline of the participants

NO.	Questions	
	Clinical nurses	Nursing managers
1.	Please share your views on the current state of nursing competencies.	What do you think of nurses’ roles and functions in the new era?
2.	What are the issues and deficiencies in your competencies?	Which competencies do you believe nurses are currently lacking?
3.	What competencies do you think nurses should develop in the future? (e.g., knowledge, skills, attitudes)	What competencies do you think nurses should acquire in the future? (e.g., knowledge, skills, attitudes)
4.	How do you think current nursing competencies will fare in the next decade?	What are your thoughts on the existing core competency training for nurses?
5.	How do you feel about nursing competency training?(e.g., teachers, duration, frequency)	

#### Research team

A strong instrument for qualitative research is the interview [26]. Two professors with backgrounds in nursing and statistics and two Ph.D. candidates with experience in qualitative research made up our research team. Professors were in charge of the study design, quality assurance, and negotiation and identification of coding themes, respectively. Ph.D. candidates were in charge of gathering and analyzing qualitative data.

### Data collection

We contacted the participants after obtaining permission from the appropriate institutions. Before the formal interviews, we explained to the participants the purpose, significance, and confidentiality of the study and assured them that they could withdraw at any time. Depending on the convenience of the participants and geographical constraints, formal interviews were scheduled via WeChat (a popular social media application in China) or face-to-face communication. All interviews were limited to 45–60 min, and participants’ nonverbal expressions were recorded using field notes.

### Data analysis

NVivo 12.0 software was used to analyze the records and create a synthetic description of the coding process. Memos and reflective notes were used as important tools for data analysis throughout the qualitative research. Two researchers were coded independently and then negotiated coding differences through group discussions until a consensus was reached. Any additional disagreements were coordinated and resolved by another professor.

The data were analyzed using a combination of inductive and deductive methods and a qualitative content analysis technique developed by Graneheim and Lundman [27]. After each interview, the recordings were transcribed promptly and subsequently imported into NVivo 12.0. After several word-by-word readings of the text, words or phrases that were meaningful to the topic were identified and then coded. The codes with the same or similar meanings were selected and categorized into the corresponding categories and subcategories. The obtained categories and subcategories are linked to the different levels of the onion model using the deductive method.

### Ethical considerations

This study was approved by the Research Ethics Committee of the First Affiliated Hospital of China Medical University (approval number: [2022]328). All participants were informed of the purpose of the study and signed a written informed consent form before data collection.

### Rigor

Four major factors contribute to the credibility of qualitative research: credibility, resonance, originality, and usefulness [27]. To ensure the study’s rigor and credibility, two researchers with qualitative research experience completed the coding task at the same time and then negotiated coding differences through group discussions until a consensus was reached, minimizing the influence of personal bias on coding. In addition, a professor on the research team will also have overall control over the disagreements of the coding. In terms of resonance, the text version formed after each coding session was spoken back to the interviewees for authenticity review. In terms of originality, based on the interview outline and the onion model, the content and hierarchy of NCC are explored, and relevant theoretical models are developed to extend the existing content. Finally, in terms of utility, qualitative data analysis allows for the expansion of new conceptual categories to improve and complement the existing NCC model, as well as provide a theoretical foundation for the future CBE.

## Result

### Demographic characteristics of the participants

**Fig. 1:**
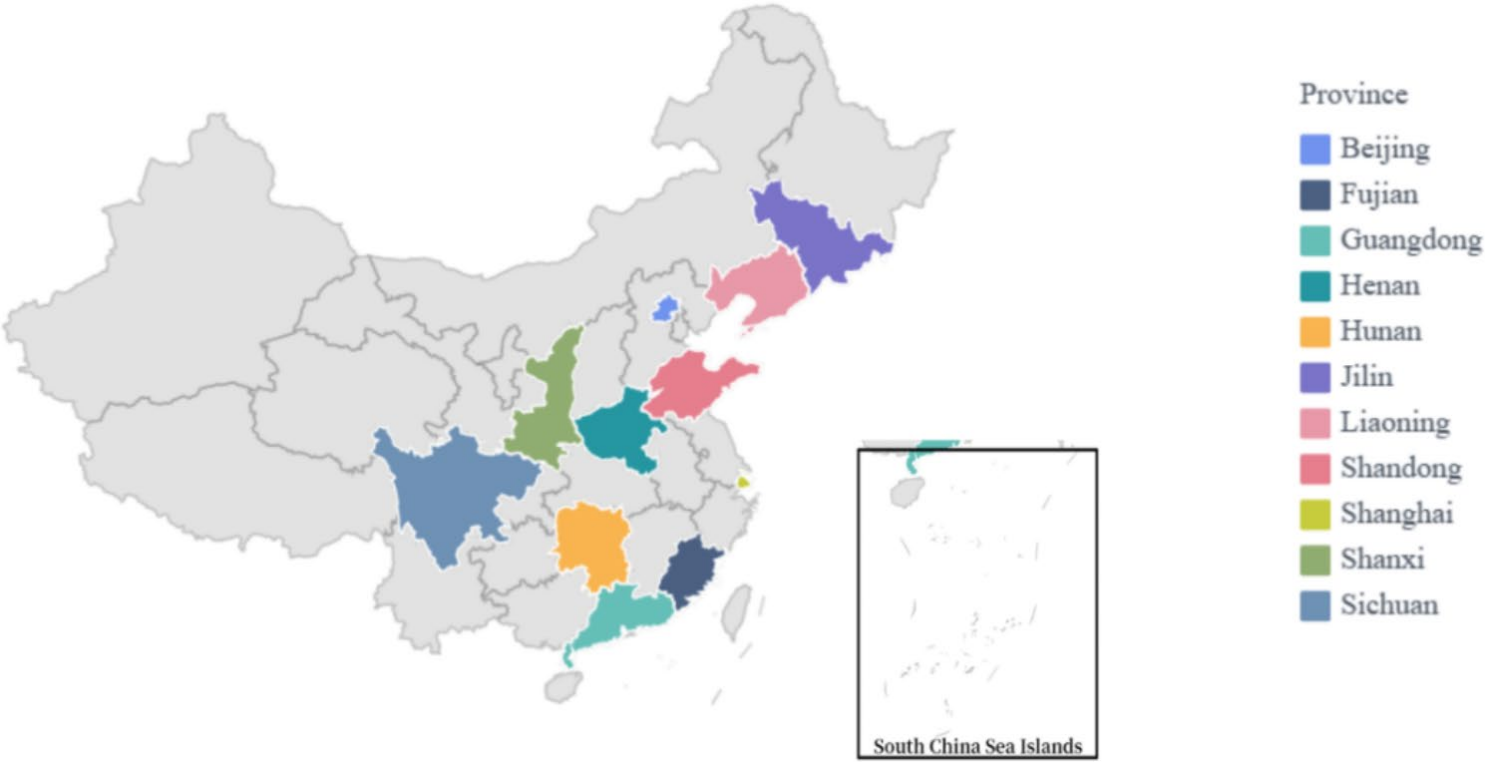
Geographical distribution of the participants

Table 2; Fig. 1 depict the participants’ demographic characteristics and geographical distribution, respectively. 20 clinical nurses and nursing managers (nurse leaders and nursing directors were included) from 11 provinces and cities were recruited to participate in semi-structured interviews. Participants included 4 males and 16 females, with an average age of (36.25 ± 9.70) and average working years of (13.00 ± 9.56) regarding.

**Table 2:** Demographic characteristics of the participants

NO.	Gender	Age	Title	Position	Education level	Working years	Section	Province
N1	Female	26	Nurse practitioner	Nurse	Master	2	Nursing department	Jilin
N2	Female	25	Nurse practitioner	Nurse	Bachelor	4	Intensive care unit	Liaoning
N3	Male	26	Nurse practitioner	Nurse	Bachelor	5	Emergency	Liaoning
N4	Male	27	Nurse practitioner	Nurse	Bachelor	4	Operating room	Liaoning
NM5	Female	35	Associate senior nurse	Head Nurse	Doctoral	11	Cardiology	Shanghai
NM6	Female	53	Senior nurse	Director of Nursing	Master	28	Nursing Department	Beijing
NM7	Female	59	Senior nurse	Director of Nursing	Doctoral	36	Nursing Department	Henan
NM8	Female	51	Senior nurse	Director of Nursing	Master	30	Nursing d department	Shandong
N9	Female	30	Nurse in charge	Nurse	Master	10	Intrarenal Dialysis	Shandong
N10	Female	31	Nurse practitioner	Nurse	Master	6	Geriatric ward	Guangdong
NM11	Female	36	Nurse in charge	Head nurse	Doctoral	10	Operating room	Hunan
NM12	Male	34	Nurse practitioner	Head nurse	Master	5	Intensive care unit	Shandong
NM13	Female	38	Associate senior nurse	Associate Director of Nursing	Doctoral	15	Nursing department	Fujian
NM14	Female	38	Associate senior nurse	Head nurse	Master	15	Pediatric general ward	Fujian
NM15	Female	34	Nurse in charge	Head nurse	Master	13	Obstetrics and Gynecology	Guangdong
NM16	Female	43	Nurse in charge	Head nurse	Master	13	Cardiology	Henan
NM17	Male	37	Nurse in charge	Head nurse	Bachelor	16	Operating room	Sichuan
N18	Female	29	Nurse practitioner	Nurse	Bachelor	8	Infection ward	Beijing
NM19	Female	45	Associate senior nurse	Head Nurse	Bachelor	23	Cardiothoracic surgery	Shanghai
N20	Female	25	Nurse practitioner	Nurse	Bachelor	5	Emergency	Shanxi

* N = nurse, NM = nursing manager

### The onion core competency model of tertiary hospitals nurses

**Fig. 2:**
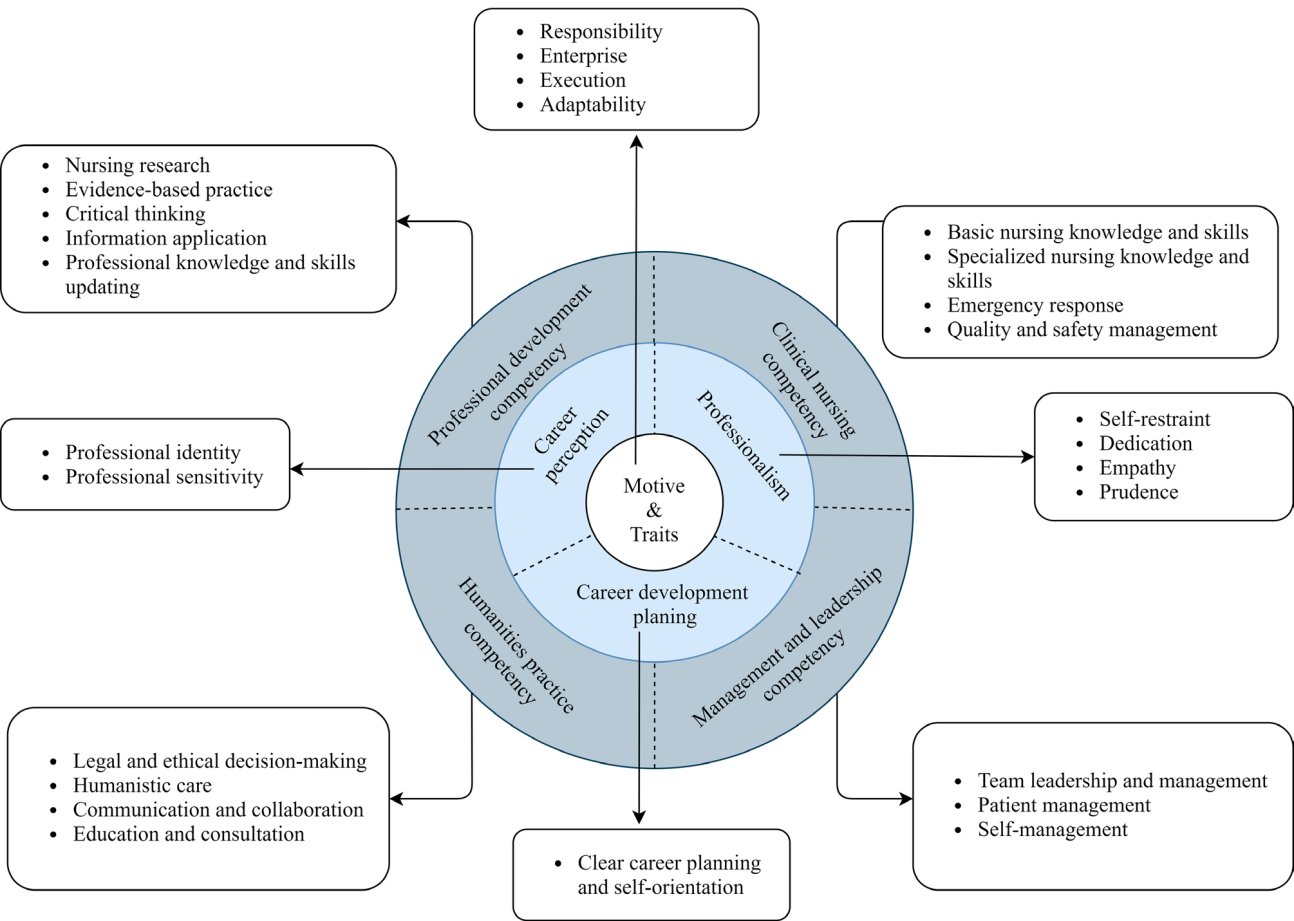
The onion core competency model of tertiary hospitals nurses

The 27 core competencies were divided into three layers through qualitative content analysis: core elements, endogenous elements, and exogenous elements, resulting in the onion core competency model of tertiary hospitals (Fig. 2) and the definition of concepts at each layer (Table 3).

**Table 3:** Definition of the concepts of core competencies for nurses in tertiary hospitals

Model element hierarchy	Theoretical element name	New element name	Definition
Exogenous elements	Knowledge, skills	Clinical nursing competency	The knowledge and operational skills necessary for nurses to perform clinical work.
		Management and leadership competency	The nurse’s management and leadership of patients, team members, and themselves.
		Humanities practice competency	The nurse’s ability and skill in using humanistic knowledge as demonstrated in clinical nursing work is the external manifestation of the nurse’s humanistic philosophy, knowledge and skills, and other factors.
		Professional development competency	The knowledge and skills that nurses should have to enhance and develop their professionalism in their clinical work.
Endogenous elements	Self-concept, attitudes, values, social roles	Professional philosophy and values	Nurses’ perceptions and attitudes toward the nursing profession.
Core elements	Motivation, traits	Motivation and traits	The personal attributes and intrinsic drivers that drive nurses to provide quality care.

#### Motivation and traits

The core layer of the onion core competency model is motivation and traits. Personal characteristics and intrinsic drivers and cores that drive nurses to develop their competency and provide quality care are referred to as motivation and traits. In this study, the majority of participants regarded a sense of responsibility as the most fundamental motivation for the core competency of nursing. Moreover, participants believed that initiative, execution, and adaptability are crucial qualities for nurses. In the following descriptions, N is for nurses, and NM is for nursing managers.

*N2: As nurses, we are first and foremost caregivers who must always be aware of our patient’s status and be accountable for their safety.*

*N14: A nurse must be a motivated individual who is capable of continuous improvement. I believe we should know what we need to improve on and stick to our objectives.*

*NM11: In my experience as a manager, nurses are very good at execution, and execution allows them to carry out medical orders as well as complete their tasks on time.*

*N18: It is especially important to learn to adapt to the change in role and thinking during the transition from student to clinical nurse.*

#### Professional Philosophy and values

The middle layer of the onion core competency model is composed of professional philosophy and values. Participants believed that nurses should have proper professional awareness. They also emphasized the importance of demonstrating professional ethics such as empathy, prudence, and dedication. In addition, having a clear career development plan and self-positioning were also considered essential by the participants.

*N10: We must be very open and tolerant, accept new things, and recognize societal changes. We need to understand what society is going through right now because only then will we be able to prepare and anticipate appropriately, and eventually realize what areas we need to improve.*

*NM17: Although there are voices in society that view nurses as subordinates to doctors, we cannot simply accept (their perception) and must make our judgments (about our professional identity).*

*NM13: In the workplace, empathy is essential. Although we have been emphasizing things like transposition and empathy, empathy is less likely to exist alongside our rational work in some process-oriented tasks.*

*N1: We must always take our work seriously, not just when the head nurse is present and then again when she leaves.*

*N4: We must exercise prudence in our work and carefully verify any information related to our patient’s conditions.*

*NM8: The choice of nursing is synonymous with dedication. Nurses are expected to possess an unwavering spirit of devotion.*

*NM7: I have noticed that some young nurses nowadays do not know what they want to do in the future. They mechanically complete their tasks in the hospital without any idea about their career development.*

#### Knowledge and skills

The outer layer is knowledge and skills. Four main components: clinical nursing competency, management and leadership competency, humanistic practice competency, and professional development competency are highlighted in this layer.

##### **Clinical nursing competency**

The cornerstone of nurses’ nursing jobs in the past, present, and future is excellent and skilled clinical knowledge and operational skills. Basic nursing knowledge and skills, specialty nursing knowledge and skills, emergency response, and quality and safety management were identified in the study.

*N20: With the application of TCM treatment in the treatment of COVID-19 pandemic, TCM is gradually gaining public recognition, and we should fully utilize the characteristics of “preventive disease treatment” in TCM care, as well as carry forward the functions of TCM rehabilitation and preventive health care.*

*N10: I believe we can do more in child health care. For example, in some countries, special people will go to the home to monitor the child’s health, rather than waiting until the disease causes irreversible damage to the child to treat it. In the future, I believe we will need to invest more in preventive health care.*

*N2: The COVID-19 pandemic has caused us a lot of trouble, and the assessment and reporting of patients need our special attention.*

*NM19: Nowadays, the majority of our patients in our department are elderly, and they all have multiple comorbidities. As a result, we must be familiar with the treatment of common conditions such as hypertension, diabetes, and cardiovascular disease, and these skills are essential.*

##### **Leadership and Management competency**

As for leadership and management competency, participants mentioned team leadership, patient management, and self-management as important components of the core competencies.

*NM11: How to tie the team to a rope, how to make everyone feel in a boat also need to have the art of. Different personalities will have to have some different management styles, humane or humane, but harsh or harsh.*

*NM14: Nurses need to have a complete understanding of the patient’s condition throughout the entire process, not only during their hospitalization but also through timely follow-up after discharge to ensure continuity of care.*

*NM8: The emergence of advanced practice nurses (APNs) is a significant global trend, and efforts made in our country to provide nurses with prescriptive authority are a crucial first step toward the creation of APNs. The nurses’ expertise in drug administration, dosage, indications, side effects, etc. is crucial to this process. With this solid foundation, they may continue to manage patients more effectively.*

*NM5: Nurses need self-management skills, including life management, emotional management, etc.*

##### **Humanistic practice competency**

Legal and ethical decision-making, humanistic care, and effective interpersonal communication are all prominent elements of humanistic practice competency. In addition, education and consultation have been identified as a crucial aspects of humanistic practice competencies.

*N4: Due to the COVID-19 pandemic, hospitals are requiring patients to have a negative nucleic acid report before they can be admitted, but if it is an emergency admission that is too late for nucleic acid, then how to make the right choice needs to cause us to think.*

*N1: We humans are thinking, we are warm, and we need to focus our efforts more on the needs of our patients.*

*NM5: You can improve communication by thinking differently and empathizing more. Even if you don’t empathize, you must learn to put yourself in the shoes of the patient to communicate and communicate more smoothly.*

*NM16: Patients come to the hospital not only to see a doctor but also for health consultations, so we need to have the ability to do health consultations and use common words.*

##### **Professional development competency**

Nursing research, evidence-based practice, critical thinking, information application, and professional knowledge and skills updating were identified in the professional development competency.

*N3: Occasionally, I may encounter new problems or methods of care in the clinic that are inconsistent with what I was taught by my instructor, and I consider going home to look up the information, but I always put it off for various reasons. In the future, I believe I will need to be able to think deeply and solve problems.*

*N2: For our promotion or performance assessment, we need to have research articles to support it. When I see others discussing research topics, I feel very envious, but I don’t know how to start or what to do.*

*NM12: We need to learn how to reflect, which means we cannot simply execute nursing tasks without any changes.*

*NM5: When we do research, we don’t just pay attention to the appearance, we want to see the common problems under the appearance of things, form a theory or model, and finally interpret the problem.*

*NM6: Because times are changing and new technologies are emerging in the field of nursing, it is critical to continue learning and updating to avoid being obsolete.*

## Discussion

Semi-structured interviews were used in this study to identify different layers and content elements of core competencies for nurses based on the onion model, which can better reveal the hierarchical structure and connotation of NCC. In addition, WHO proposes that the world is currently witnessing an unprecedented contribution of nursing to universal health coverage, and there is a need to invest in capacity building [28]. In the next decade, there must be a rapid development of nursing education to cultivate nurses capable of addressing future challenges [29]. The NCC identified in this study is based on the genuine needs of nurses under China’s new era of health development strategy. Using the onion model, the NCC has been divided into three distinct levels based on their level of difficulty, providing a reference for managers in different countries to better understand the requirements of NCC at different levels and develop the nursing profession’s capabilities.

**Core Competencies of nurses under the new healthy development strategy**.

The most central level of need in the NCC is motivation and traits, which are the intrinsic, difficult-to-measure aspects of people that are the most difficult to uncover and change. It is undeniable that nurses’ sense of responsibility for the safety of their patients’ lives remains the nursing profession’s most fundamental mission commitment. Nurses’ competency to continually improve and develop is driven by enterprise, and both execution and adaptability are traits possessed by a highly qualified nurse.

Professional philosophy and values are both the foundation of nursing practice and the cornerstone of nurses’ ability to provide high-quality nursing care [30]. Perceptions of the nursing profession directly influence individual behavior and performance, and by being sensitive to changes and trends in the nursing field, they can have a clearer understanding of their career plans. Furthermore, some participants mentioned that choosing to nurse is a choice of dedication. Dedication, as a professional spirit rooted in the nursing profession, is at the heart of a nurse’s profession [31].

Clinical nursing competency, leadership and management competency, humanistic practice competency, and professional development competency are the most accessible and cultivated competencies. The majority of this related knowledge and skills are similar to the competencies in the previously developed core competency framework for nurses in China [6]. In this study, the real needs of nurses and reflections on competency development were included, and these differences and additions may reflect the new preferences and needs of nurses in the context of times and social developments that are shaped by the various values and strategic goals of society and the health care system. Nurses require a diverse set of skills to provide quality care to patients in an ever-changing clinical environment. Because of the development of nursing specialization in China, common knowledge and nursing skills in specialties such as hospice care, chronic disease management, TCM, and maternal and child health must be gradually learned and applied in addition to basic nursing knowledge and skills. In the same way, a multidisciplinary understanding of the disease, such as anatomy, physiology, biochemistry, and pathology, is better suited to providing quality patient care. Even though not all participants held leadership positions, the majority of participants mentioned this important competency. Leadership and management competency is not unique to nursing managers, in addition to administrative competencies, nurses’ leadership can provide quality care to patients by infecting the surrounding healthcare professionals and patients through their own professional and personal charisma [32]. All nurses play an important role in leadership and are encouraged to learn more about it in the ever-changing healthcare field [33, 34].

Person-centered care and respect for the patient’s feelings and needs as a “human” are more important in the new health development strategy, and humanistic practice competencies, which include legal and ethical, interpersonal communication, humanistic care, and other related competencies, are being raised by an increasing number of nurses. Surprisingly, almost all nurses mentioned the difficulty of communication. One of the causes of communication barriers could be a mismatch in information [35]. The question of how to communicate effectively with patients and colleagues in a high-stress clinical situation remains unanswered. The biomedical model, which focuses on diagnosis and treatment, continues to heavily influence how healthcare professionals view patient care in most clinical settings in China. Nurses are more likely to be concerned with the patient’s physical condition and less concerned with psychological care, which contradicts the holistic nature of care to some extent [36, 37]. The importance of communication in humanistic care cannot be overstated; however, good interpersonal communication in clinical situations necessitates a significant amount of effort, and empathy and patience may be important ways to achieve comfortable and acceptable communication.

Professional development skills are the knowledge and skills that nurses should have to improve and develop their clinical professionalism. It is not only a technical tool, but it is also the most effective method of obtaining scientific information, which is a necessary skill for nurses. Many nurses in research have suggested that the ability to use information is important. The rapid advancement of information technology is gradually transforming the medical and nursing care delivery model [38]. Nurses should fully utilize information technology tools to expand their service areas to adapt to this new model of care. Furthermore, the shift in mindset aids in the development of competencies and increases motivation for behavior change. As a result, the future focus and difficulty will be how to fully use critical thinking in nursing decision-making and train the shift in mindset [39].

**Cultivation and development of core competencies of nurses in tertiary hospitals**.

Both the “Healthy China 2030” Strategy and the 20th Communist Party of China Report advocate for a people-centered approach to health maintenance in all aspects and cycles. In light of the people’s growing health needs, the development of nursing capabilities must be integrated with the current social context, expanded the scope of nursing services, and integrated with the people’s health needs. As patients’ individual needs, disease complexity, and treatment modalities evolve, the development of nurses’ core competencies must become more varied depending on the stage.

According to the findings of this study, the majority of nurses believe that their competencies are still lacking in many ways and that they should constantly improve and update their professional knowledge and skills to meet the health needs of patients. The nurses’ ability to integrate and apply their knowledge is limited, and they have a fixed mindset, making it difficult to apply their learning. This could be due to a mismatch between what nurses learn in school and the clinic, as well as a gap between what they learn in training and clinical practice. Furthermore, the supervising teacher influences the working style and attitude of the new nurses and may have a significant impact on their professional perceptions [40]. Moreover, not all nurses consider their competence development deeply, and they are frequently accustomed to accepting passive learning, settling for the status quo, and being less self-driven. In China, the Confucian educational ideology handed down through history, as well as the modern education system that mimics the former Soviet Union’s classroom style, have severely limited the development of students’ creative ways of thinking [41, 42], therefore, a change in the traditional educational model should be considered. Competency-based education for nurses may be an effective way to enhance the thinking and developmental skills of nurses [43]. Competency-based education (CBE) broadens the theoretical and practical training content, and this study has identified three levels of nurses’ core competencies using the onion model. Going forward, CBE will be an important approach for enhancing nurses’ comprehensive competencies, such as problem-solving and critical thinking, and improving the quality of nursing services [44–46].

## Conclusion

Based on the onion model, three layers of core competencies for nurses in Chinese tertiary hospitals were identified. Under the new health development strategy, nurses need not only comprehensive knowledge and skills such as clinical nursing competencies, management and leadership competencies, humanistic practice competencies, and professional development competencies but also positive professional perceptions and values. More importantly, strong motivation and good traits are significant internal motivators for nurses to improve their competencies. The different layers of NCC identified by the study can serve as a theoretical reference for nursing managers to better understand nurses’ various levels of competency and conduct CBE based on their layers. In future studies, relevant measurement tools can be developed to assess and identify weaknesses in NCC based on the model, and then construct targeted competency enhancement strategies based on assessment results.

This study has one limitation: using the onion model as a deductive method for data analysis may result in some biases. To minimize the potential bias, inductive and deductive methods were used to analyze the data.

## Data Availability

The datasets used and/or analyzed during the current study are available from the corresponding author upon reasonable request.

## Abbreviations

COREQ: Consolidated criteria for reporting qualitative research
CBE: Competency-Based education
NCC: nursing core competency
